# Intermolecular and Electrode-Molecule Bonding in a Single Dimer Junction of Naphthalenethiol as Revealed by Surface-Enhanced Raman Scattering Combined with Transport Measurements

**DOI:** 10.1021/jacs.3c02050

**Published:** 2023-07-12

**Authors:** Kanji Homma, Satoshi Kaneko, Kazuhito Tsukagoshi, Tomoaki Nishino

**Affiliations:** † Department of Chemistry, School of Science, Tokyo Institute of Technology, 2-12-1 W4-10 Ookayama, Meguro-ku, Tokyo 152-8551, Japan; ‡ International Center for Materials Nanoarchitectonics (WPI-MANA), National Institute for Materials Science (NIMS), 1-1 Namiki, Tsukuba, Ibaraki 305-0044, Japan

## Abstract

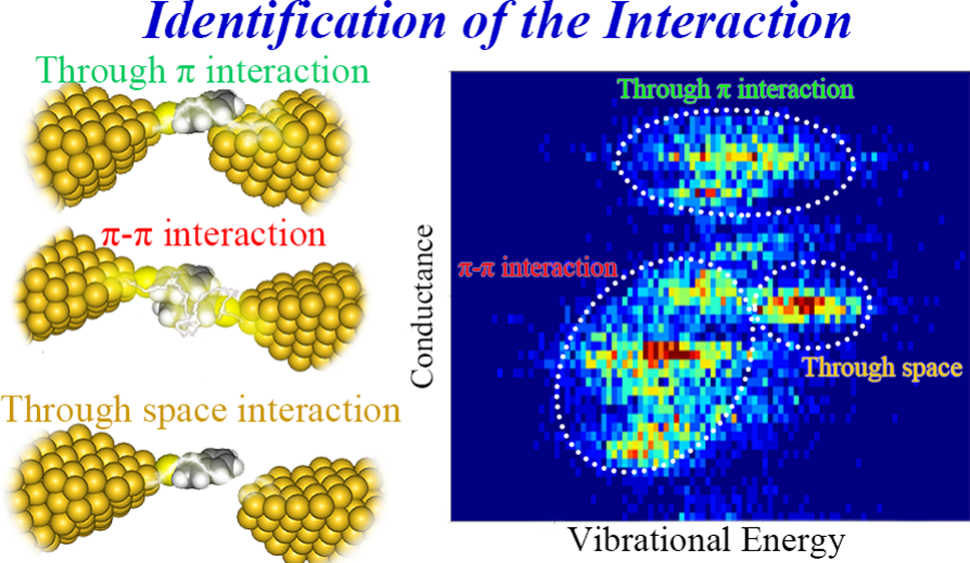

Electron transport through noncovalent interaction is of fundamental and practical importance in nanomaterials and nanodevices. Recent single-molecule studies employing single-molecule junctions have revealed unique electron transport properties through noncovalent interactions, especially those through a π–π interaction. However, the relationship between the junction structure and electron transport remains elusive due to the insufficient knowledge of geometric structures. In this article, we employ surface-enhanced Raman scattering (SERS) synchronized with current–voltage (*I*–*V*) measurements to characterize the junction structure, together with the transport properties, of a single dimer and monomer junction of naphthalenethiol, the former of which was formed by the intermolecular π–π interaction. The correlation analysis of the vibrational energy and electrical conductance enables identifying the intermolecular and molecule–electrode interactions in these molecular junctions and, consequently, addressing the transport properties exclusively associated with the π–π interaction. In addition, the analysis achieved discrimination of the interaction between the NT molecule and the Au electrode of the junction, i.e., Au−π interactions through-π coupling and though-space coupling. The power density spectra support the noncovalent character at the interfaces in the molecular junctions. These results demonstrate that the simultaneous SERS and *I*–*V* technique provides a unique means for the structural and electrical investigation of noncovalent interactions.

## Introduction

Noncovalent interactions play a decisive role in a self-assembly process in organic, inorganic, and biological materials.^1−4^ The combination of the interactions enables sophisticated structural design for a wide range of nanomaterials and construction of superior systems for molecular recognition.^2−5^ In particular, electron transport through the π–π interaction in π-conjugated molecular systems has attracted significant attention to build novel electronic materials taking advantage of the unique delocalized electric states intrinsic to the constituent molecules.^6,7^ For example, the efficient long-range charge transport via π–π interactions makes DNA promising building blocks in ultrasmall electronic materials.^7^ The availability of the quantum interference effect^8−14^ to control the electron transport gives the π-conjugated systems an additional advantage in electronics applications. Recently, single-molecule techniques were adopted for the electron transport of molecular junction composed of a single π-dimer in order to gain fundamental insights into the transport properties.^12−18^ Indeed, the formation of the single π-dimer junction was confirmed using the oligo-phenylene-ethynylene (OPE) derivatives by the electrical measurements,^16,17^ and it was shown that for OPE derivatives, the electronic structure of a π-dimer was significantly modulated upon applying mechanical stress.^13^ It was also found that the electron transport through a π-dimer exhibited unique noise characteristics.^15^ In the latter example, the conductance value of the junction was enhanced in the antiparallel alignment of the dipole moment in the azulene-based dimer.^15^ These studies have brought substantial fundamental understanding in terms of the electron transport properties at the single-molecule level, highlighting the unique behavior of the single-dimer junction as compared with the monomeric counterpart. In contrast, knowledge about structures of the π-dimer junction has remained elusive since the geometric structures of molecular junctions cannot be generally deduced from the electron transport measurements. This hampers the evaluation of the electron transport properties in light of the structure-properties relationship. In addition, the lack of structural information could even lead to situations where experimentally determined transport properties for dimer junctions contain contributions from those for the monomer junctions, as is revealed in the present work.

**Figure 1:**
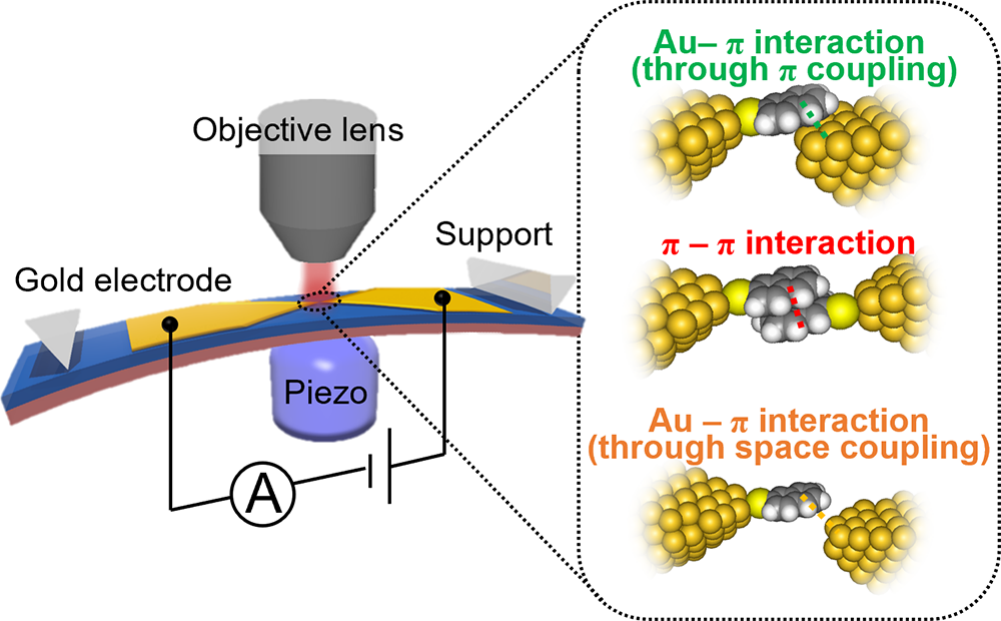
Schematic illustration of the mechanically controllable break-junction (MCBJ) combined with surface-enhanced Raman scattering (SERS) measurements. The inset represents the possible interactions involved in the formation of the NT molecular junction.

Therefore, structural characterization is imperative in pursuit of developing functional single-dimer junctions. Identification of bondings at the intermolecular interface (π–π interaction in the case of the π-dimer junctions) and at the metal-molecular interfaces (either through-bond or through-space coupling) is of particular importance given the decisive role played by the interface structures in the electronic properties of the molecular junctions.^19,20^ SERS measurement synchronized with the electron transport measurement provides a unique means to reveal the molecular structure at the single-molecule level.^21−25^ We have applied this technique to the single-molecule junctions and successfully revealed their interfacial structures.^23,26−28^ In this research, we investigate a molecular junction bridged with a single naphthalenethiol (NT) molecule or with a single π-dimer of NT (Figure 1). In situ conductance and SERS measurement based on the MCBJ technique detect changes in the vibrational energy correlated with the junction conductance, and evolution of the junction structure was deduced. The conductance dependence of the vibrational energy enables identification of the bondings in the molecular junctions: intermolecular π–π interaction, through-π coupling, and through-space coupling (Figure 1). Importantly, the single dimer junction with the intermolecular π–π interaction and the single-molecule junction with the through-space coupling are indistinguishable from each other by the electrical measurement alone due to considerable overlap of their conductance values. The flicker noise analysis of the conductance fluctuation supports electron transport through the bonding in the molecular junctions found in the simultaneous SERS and MCBJ measurements, although discrimination between different bondings cannot be attained by the noise analysis. The density functional theory (DFT) calculation of the vibrational energy and electron-transport properties corroborates the experimental findings. The present study based on the simultaneous optical and electrical measurement provides in depth structural characterization of the molecular junction containing the single π-dimer, raising the possibility of aromatic molecules as electric nanomaterials for molecular electronics.

## Experimental Section

The molecular junction was fabricated by the MCBJ technique (Figure 1).^9,23,27−30^ The fabrication process can be found elsewhere.^20−23^ Briefly, the gold electrode was fabricated on the SiO_2_ layer on the phosphor bronze plate coated by polyimide using the electron beam irradiation and lift-off process. The O_2_ dry-plasma etching removed the polyimide under the central part of the gold electrodes, fabricating the free-standing structure. The 1 mM ethanol solution of NT was dropped on the substrate, cast, and dried under ambient conditions, allowing for the formation of a self-assembled monolayer on the gold electrode. The substrate on the three-point-bending configuration was bent by the pushing rod and bending was controlled by the piezo element. The piezo element halted during the SERS and conductance measurements in the conductance region of the molecular junction (10^–1^–10^–5^*G*_0_). The conductance was monitored via a data acquisition system (NI-4661, National Instruments corp. The US) with a bias voltage of 200 mV. The SERS was measured by a Raman spectrometer (Nanofinger 30A, Tokyo Instruments, Japan). The *I*–*V* curves were measured every 200 ms in the range of ±1 V The sampling rate was 100 kHz. The 785 nm laser with a power of 10 mW was irradiated on the central part of the substrate via 50× objective lens. The interval of the data acquisition of Raman spectra was 200 ms. SERS and conductance signals were connected via the trigger signal from the spectrometer.

The vibrational energy and peak intensity of SERS spectra were determined by fitting using the Lorentz function.^31^ The conductance was calculated from the average of the slope in the *I*–*V* curves in the region of 10–100 mV. We estimated the flicker noise following the previous papers (Supporting Information S1 and S2).^19^ The time course of the conductance during each SERS measurement was converted to the noise power (*S*_n_) by calculating power spectral density (PSD) using the fast Fourier transformation (FFT). The flicker noise (*S*_FN_) was then calculated by integrating the PSD in the region of 100–1000 Hz and normalized by the averaged conductance. The geometry of the Au/NT/Au junction was constructed by the Virtual NanoLab-Atomistix ToolKit package (ver. 2014.2), which relies on DFT combined with the nonequilibrium Green’s function method.^32^ The optimization and transport calculation were performed by the Perdew–Burke–Ernzerhof generalized gradient approximation exchange-correlation energy functional, considering the π–π interaction at each separation distance of the two gold electrodes. We simulated 33 and 58 transmission spectra for the monomer and dimer junctions, respectively, to investigate the conductance behavior throughout the geometries of the NT junctions from the connected to disconnected states. The vibrational energy of the NT junction was calculated by the Gaussian 16 after the geometrical optimization.^33^

The geometry of the junction was optimized by the DFT method of CAM-B3LYP with Grimme’s D3BJ empirical dispersion correction to include the effect of the dispersion force.^34^ The basis function was LanL2DZ for gold and 6-31G(d) for carbon, hydrogen, and sulfur atoms. Details can be found in Supporting Information S3.

## Results and Discussion

**Figure 2:**
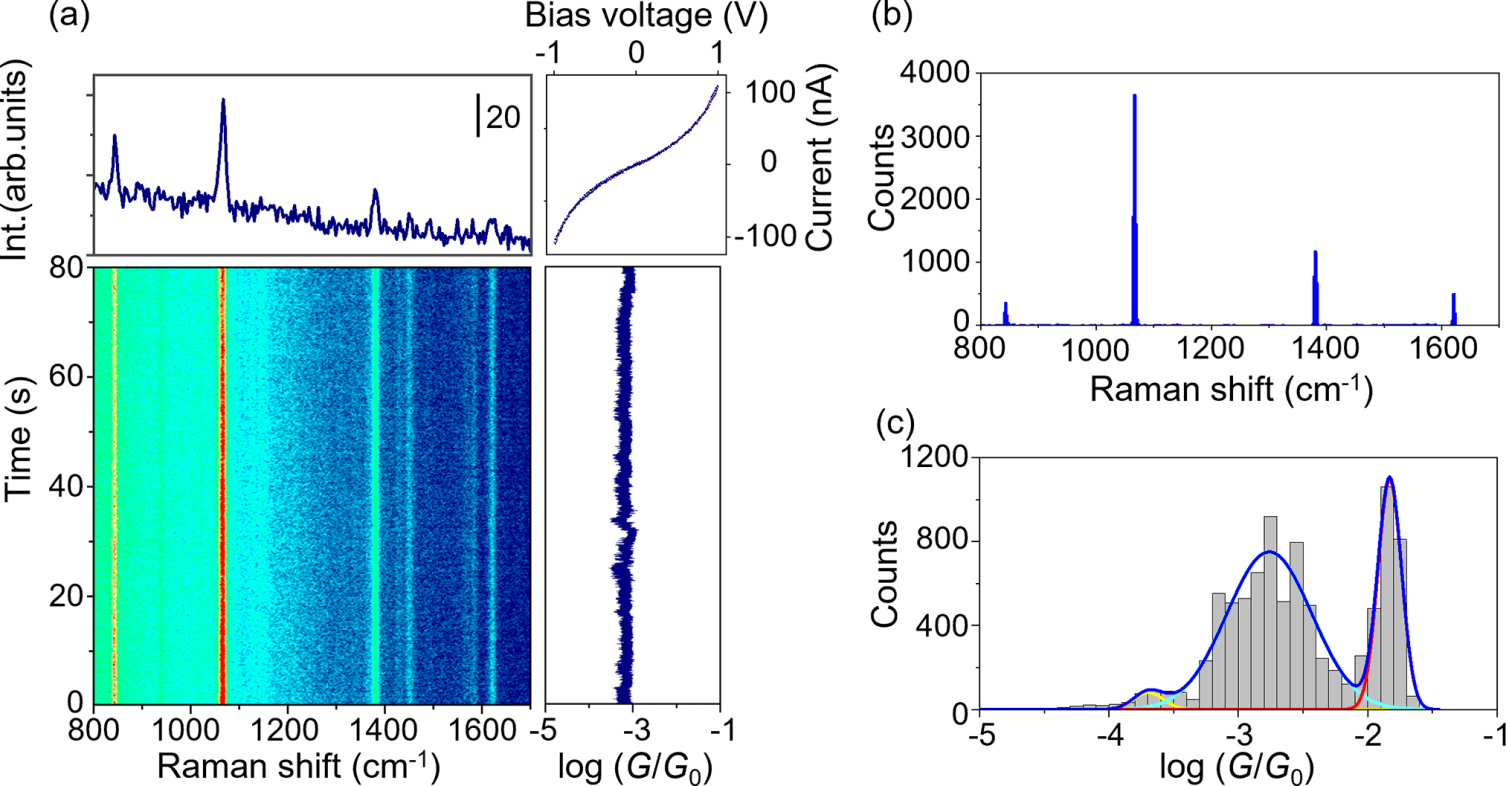
SERS and electric conductance measurement for the NT molecular junctions. (a) Time course of the conductance (*G*) and SERS intensity, accompanied by the typical SERS spectrum and simultaneously acquired current–voltage (*I*–*V*) curves. (b) Histogram of the Raman shift of the observed peak obtained by the fitting with the Lorenz function. (c) Histogram of Raman shift and conductance was constructed from 8985 SERS spectra and *I*–*V* curves.

Figure 2a represents the trajectory of SERS intensity and conductance obtained by the MCBJ measurements using the NT-modified electrodes, accompanied by the typical SERS spectrum acquired at a junction conductance of 1 m*G*_0_ (*G*_0_ = 2*e*^2^/*h*). This conductance range was chosen based on separate electric measurements using the MCBJ technique to ensure the formation of the NT molecular junctions (Supporting Information S4). As can be seen in the conductance trace in Figure 2a, persistent formation of the molecular junction was observed. The relatively long junction lifetime is ascribed to the highly stable electrodes in the MCBJ setup.^23,35,36^ In addition, the electrodes were held stationary during the SERS and *I*–*V* measurements, which further stabilizes the molecular junction.^23^ For statistical robustness, we analyzed 30 trajectories including 8985 SERS spectra and *I*–*V* curves. The histogram of the energy of the vibrational modes observed in the SERS spectra shows prominent peaks at 1067 and 1380 cm^–1^ originating from the ring breathing mode and CH bending mode of NT, respectively, together with the other vibrational modes also assigned to NT molecule (Figure 2b, Supporting Information S5). The enhanced SERS signal observed in the typical conductance range of the molecules supports the formation of the molecular junction (Supporting Information S6), representing the presence of the NT molecule at the nanogap.^21−23,28,37−39^ In addition, the nonlinear behaviors in the *I*–*V* curves simultaneously measured with the SERS spectra demonstrate that the NT molecule is involved in the electron transport.^23,26^ The synchronized SERS signal with the *I*–*V* response represents the formation of the molecular junction. The conductance histogram from the *I*–*V* responses showed two conductance states: high conductance state (H state) around 10^–1.8^*G*_0_ and the low conductance state (L state) around 10^–2.8^*G*_0_ accompanied by the satellite peak (Figure 2c). The observed conductance values agreed with those observed by the standard break junction method (Supporting Information S4). The possible structures for the observed conductance states are the NT single-molecule junction, i.e., the Au/NT monomer/Au junction, and the molecular junction accommodating a single NT dimer with the intermolecular π–π interaction, i.e., the Au/NT dimer/Au junction. According to the previous study, single-molecule junctions in which the molecule interacts with the metal electrodes via the direct π-bonding tend to show high conductivity,^9,40^ while the typical conductivity of single π-dimer junctions is around 10^–3^*G*_0_.^14,15^ The analogy with the previous studies indicates that the H state originates from the Au/NT monomer/Au junction, while the L state originates from the Au/NT dimer/Au junction.

The following detailed analyses of the simultaneous SERS and *I–V* results demonstrate that this conventional interpretation holds only partially true at least for the present junction, and careful structural characterization is necessary for the π-dimer junctions.

**Figure 3:**
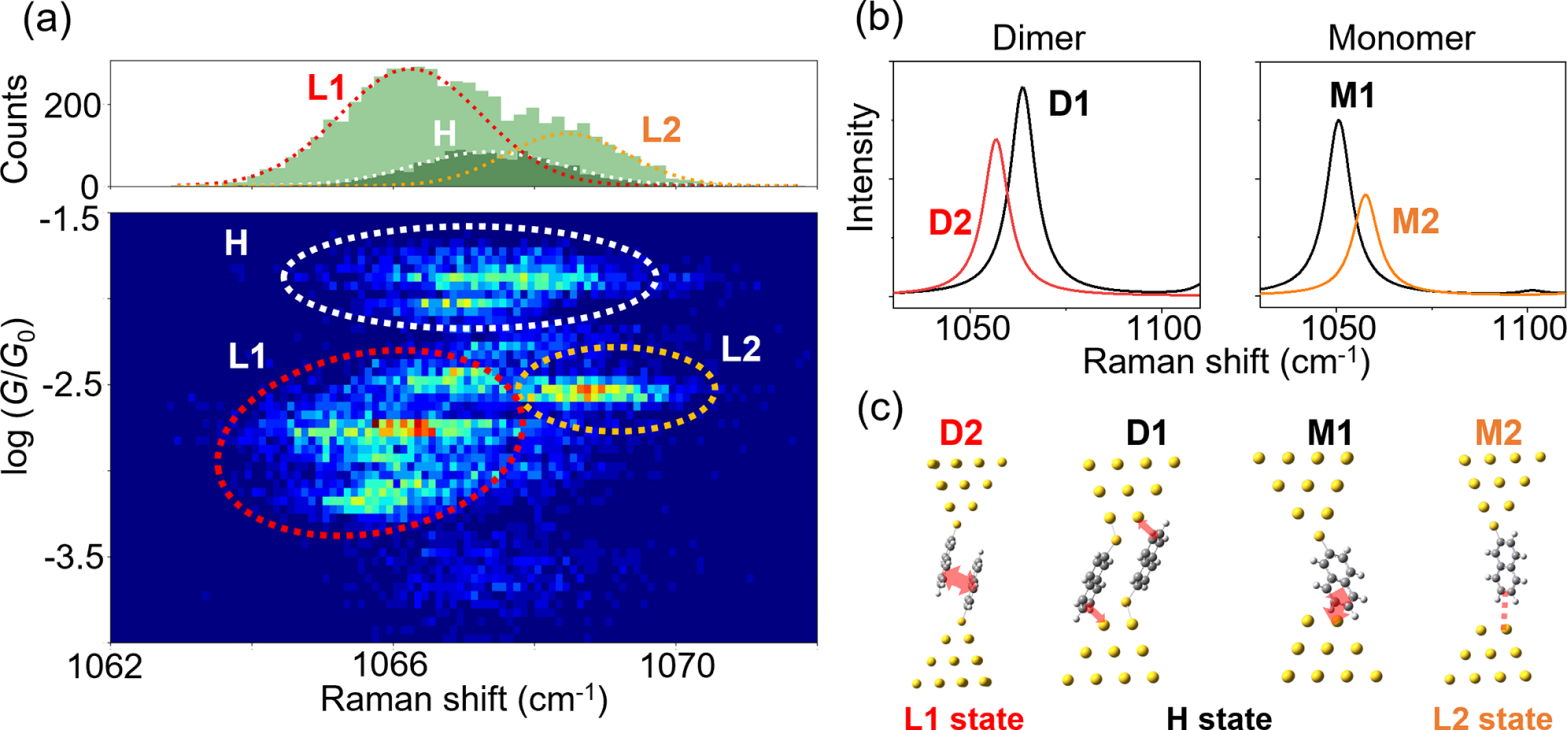
Structural analysis of NT molecular junctions by SERS spectra. (a) Two-dimensional (2D) histogram for Raman shift and conductance, along with the one-dimensional histogram of Raman shift. (b) Calculated spectra for the ring breathing mode. (c) Optimized structural models of the NT molecular junctions. Their assignments are also shown.

To elucidate the configuration of the NT junction, we analyzed the relationship between vibrational energy and conductance. Figure 3a shows a two-dimensional histogram between conductance and vibrational energy of the ring breathing mode of the NT molecule in the junction. Apparently, three states are recognized in this plot. The center of the distribution for each state corresponds to the time-averaged vibrational energy and conductance of the junction with the most probable structure of the metastable state. The distribution of the vibrational energy was located around 1067 cm^–1^ for the H state, while two components appeared for the L state: i.e., around 1066 cm^–1^ (L1) and 1068 cm^–1^ (L2). Though the difference in the vibrational energy is small, the statistical correlation analysis clearly resolves three domains. The coexistence of the two states in the L state region is corroborated by the CH bending mode observed around 1380 cm^–1^ (Supporting Information S7). The L1 and L2 states cannot be distinguished from each other solely by the conductance measurement, which underscores the importance of simultaneous spectroscopic studies in structural characterization of molecular junctions. We constructed representative model structures of the NT molecular junctions as shown in Figure 3c. The model junctions contain either the NT dimer (D1 and D2) or the single NT molecule (M1 and M2).

In addition to the number of constituent molecules, different molecule/electrode interactions were taken into account, i.e., either strongly (D1 and M1) or weakly (D2 and M2) coupled cases, to capture the energetic shifts of the vibrational modes of the molecular junctions for the L1 and L2 states. In the strongly coupled cases, the naphthalene ring directly interacted with the gold electrodes (through-π coupling in Figure 1). This coupling scheme causes high electric conductance, as confirmed by the transport calculations discussed below, and consequently, the D1 and M1 models are assigned to the H states. The reduction of the coupling with the electrodes in the D2 and M2 structures (through-space coupling in Figure 1) indicates a conductance decrease (see below for the transport calculations). Thus, these structures are attributed to either the L1 or L2 states. Then, the vibrational spectra were analyzed for further assignments. DFT calculations were performed to predict the vibrational energies for the molecular junctions in Figure 3c, and the calculated spectral features were compared with those found in the 2D histogram (Figure 3a). The 2D histogram reveals the vibrational energy of the NT molecular junctions with the statistically most probable structures (see above), and thus this statistical analysis justifies the comparison between the experimental data with the structurally optimized models illustrated in Figure 3c. The calculation revealed that for the dimeric junctions the vibrational energy of the D2 structure decreased compared with that of the D1 structure, which is assigned to the H state (Figure 3b). In contrast, increased vibrational energy was found for the M2 structure in comparison with the M1 structure in the H state (Figure 3b). These vibrational shifts are in nice qualitative agreement with the experimental results in Figure 3a and lead to the assignment that the molecular junctions with the structures represented by the D2 and M2 models were responsible for the L1 and L2 states, respectively.

The origin of the vibrational energy shift is interpreted by the charge transfer and the interaction of the naphthalene ring.^27,30^ It has been shown that the charge transfer induced by molecular adsorption on a metal surface reduces the vibrational energy of the ring breathing mode.^37^ This mechanism explains the difference in the monomer junctions: the vibrational energy is smaller for the strongly coupled case (M1) than for the weakly coupled counterpart (M2). In the case of the dimer junction, electron transfer between NTs by the formation of the dimer has been known to decrease the vibrational energy of the ring breathing mode.^38^ This fact rationalizes the decrease in the vibrational energy for the D2 structure compared with the M2 structure. In addition, we calculated the energy of the NT dimer depending on the displacement of the dimer (Supporting Information S8).

The binding energy of the NT dimer was estimated to be around 350 meV agreeing with the previous studies of the π stacked dimers,^15,38,41^ supporting the existence of the NT dimer in the breaking process of the NT molecular junction. It is thus concluded that the π-dimer junction and monomer junction weakly coupled with the electrode constitute the L1 and L2 states, respectively, in Figure 3a. This finding has an important implication for the study of single π-stacked molecular junctions. The conductance histogram in Figure 2c exhibited the two conducting states, and the conventional interpretation is that the L state originates from the π-stacked junction. However, the present study demonstrates that the weakly coupled single-molecule junction coexists with the π-dimer junction in the low-conducting states.

**Figure 4:**
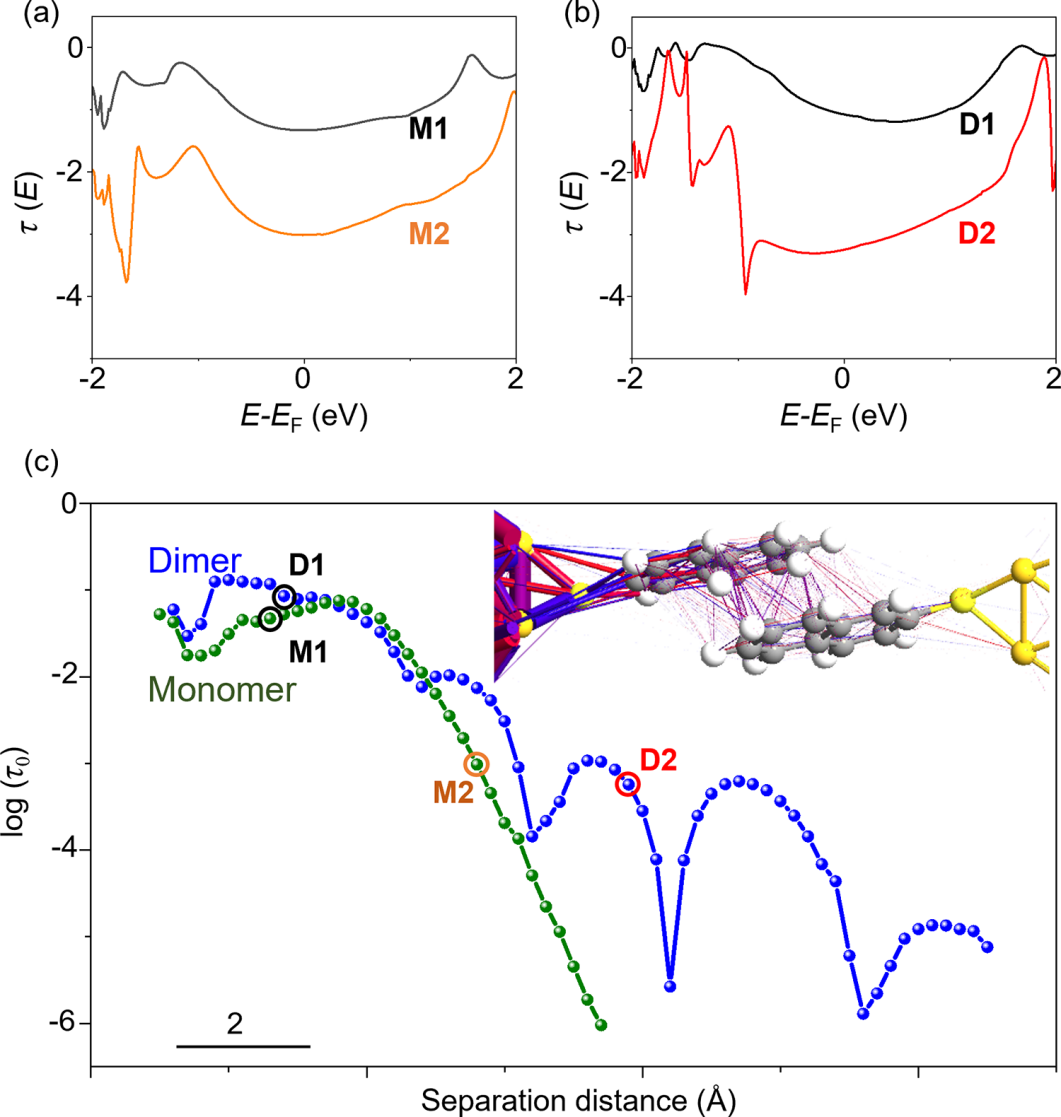
Simulation for the electron transport properties. (a) Transmission spectra of NT monomer junction with M1 and M2 structures. (b) Transmission spectra of NT dimer junction with D1 and D2 models. (c) The separation distance dependence of the transmission coefficient at the Fermi level (τ_0_). The green and blue curves indicate the monomer and dimer junctions, respectively. The points corresponding to the M1, M2, D1, and D2 structures were marked by circles. The inset illustrates electron transport trajectory of the NT dimer junction with the D2 structure at the zero-bias voltage. Colored lines represent the direction of the electron transport. Blue lines represent transport in forward direction, while red one represents that in the opposite direction.

To support the models proposed above, we then evaluated the electron transport through the NT molecular junction. The transmission curves for the molecular junctions of the NT monomer (M1 and M2) and dimer (D1 and D2) adopting the configuration depicted in Figure 3c are displayed in Figure 4a,b, respectively. These curves show that the highest occupied molecular orbital (HOMO) and the lowest unoccupied molecular orbital (LUMO) of NT are located around −1 and 2 eV, respectively, indicating that the electrons dominantly transport via the HOMO in accordance with the previous report.^42^

Furthermore, the calculated transport trajectory demonstrates that in the junction with the D2 structure the electron transmission occurs via π–π interaction through hybridization of the NT HOMOs (Figure 4c inset). The conductance values of the M1 and D1 models were estimated to be 10^–1.3^*G*_0_ and 10^–1.8^*G*_0_, respectively, while those of the M2 and D2 models were calculated to be 10^–3.0^*G*_0_ and 10^–3.2^*G*_0_. These conductance values qualitatively agree with those observed in the experiments (see Figure 3a). Quantitatively, the differences in the conductance values between the four models found in the calculations were larger than the differences observed in the experiments, as was commonly encountered in comparing the experimental results of single-molecule electron transport with the calculations.^43−45^ Taken together, the computational results corroborate the structural identification summarized in Figure 3c. In order to compare the calculated transmission spectra with the experimentally observed transport properties, we additionally calculated transmission spectra by varying the gap width between the electrodes for the monomer and dimer junctions. The transmission coefficients at the Fermi level (τ_0_) extracted from the individual transmission spectrum were plotted as a function of the gap width (Figure 4c), which represents the conductance traces. At the small distances, which correspond to the M1 and D1 models, the transmission coefficients of the monomer and dimer junctions nearly coincide with each other. On the other hand, at the longer distances, associated with the M2 and D2 models, the transmission coefficient shows distinct differences between the monomer and dimer junction. The conductance of the monomer junction steeply decreases with increasing the distance, while the conductance of the dimer junction gradually decreases with multiple dips. This different behavior for the monomer and dimer junctions can be found in the experimental 2D histogram in Figure 3a as the width of the conductance distributions. The conductance of the L2 state, associated with the M2 structure, was narrowly distributed, and the conductance distribution of the L1 state, caused by the D2 structure, was widely distributed. In addition, it is noticeable that the transmission of the dimer junction shows periodical dips that can be attributed to the destructive quantum interference.^11,13,46−48^ When the molecular orbitals overlap with the opposite symmetry, the conductance significantly decreases.^13,46^

Although this destructive interference effect cannot be completely distinguished from the disconnected junction in our conductance measurement, the vibrational energy behavior indicates the presence of the dimer junction with the destructive conductance states. The broad distribution of the vibrational energy in the range of the 10^–4^–10^–3^*G*_0_ indicates that at least two components contribute to this conductance region (Supporting Information S9). The smaller vibrational energy probably corresponds to the dimer molecular junction, while the larger vibrational energy indicates the weakly bound NT junctions.

**Figure 5:**
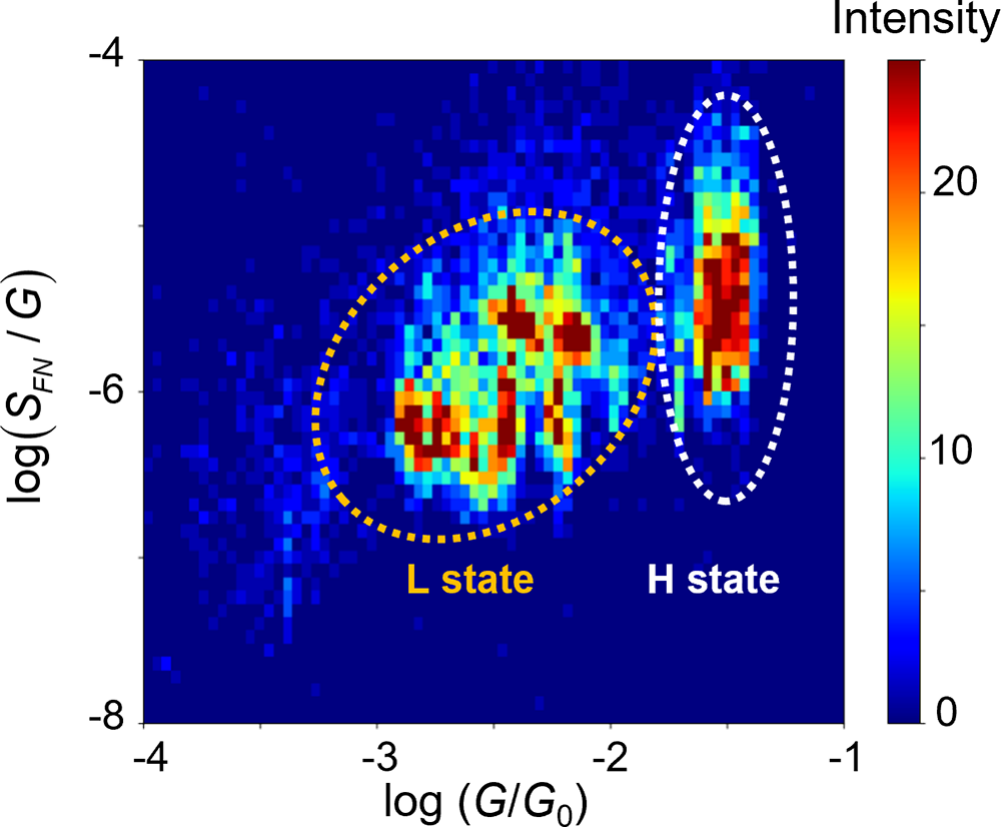
2D histogram of the flicker noise power (*S*_FN_) and conductance. White and orange circles indicate the L and H states, respectively.

The characteristics of the molecule-electrode interactions in the NT molecular junctions were investigated by the flicker noise behaviors.^14,19,20^ We calculated PSD from the time course of the conductance of the molecular junctions by FFT (Supporting Information 1). The noise power density (*S*_n_) follows a power law with the frequency (*f*), i.e., *S*_n_ ∝ *f*^–1.1±0.4^ in agreement with the typical flicker noise values observed for molecule junctions.^19,20,49^ The enhanced SERS is a 2D histogram of the noise power (*S*_FN_) normalized by the averaged conductance against the averaged conductance (*G*_AVE_), as shown in Figure 5. The histogram shows sharp distribution for the H state associated with the NT monomer junction. The DFT calculations on the junction structure indicate that the monomer junction exhibits a very limited range of the conductance due to facile destabilization of the junction upon changes in the gap width between the electrodes (Supporting Information S10). This limitation significantly diminished the correlation between the conductance and *S*_n_. In fact, the correlation analyses revealed the correlation factor of 0.1 for the H state, while the corresponding value reached 0.4 for the other L state. The nearly uncorrelated relationship hampers precise determination of conductance dependency of flicker noise, arising from the specific character of Au−π bond (details were discussed in Supporting Information S2).^15,50,51^ In stark contrast, for the L state, the noise power follows power law and scales with *G*^1.6^. The NT monomer and dimer junctions contain a variety of interfaces (see Figure 3c): the chemisorption interface via Au–S bonds, and the physisorption interfaces between the NT molecule and the electrode and between the NT molecules within the π-dimer. The noise powers of molecular junctions with the former and the latter interfaces show scaling of *G* and *G*^2^, respectively (Supporting Information S2).^19^ In contrast, it has been found that the noise power scaling becomes a nearly intermediate value, i.e., *G*^1.6^, for the junction with both the chemisorption and physisorption interfaces.^14,19^ The exponent for the L state derived from Figure 5 accords with this value and, therefore, demonstrates that in the NT junctions in the L state the electron transport involves not only the Au–S bond but also the noncovalent interaction. This picture supports the structural assignment made based on the SERS studies (Figure 3) that the NT junctions with the D2 and M2 structures constitute the L state. However, these junctions are equivalent in terms of the interfaces, since they contain a common chemisorption component at the Au–S interfaces and physisorption component either within the NT dimer in the case of D2 or at the NT/electrode contact for M2. Thus, unequivocal structural identification cannot be attained by the flicker noise analyses, which again highlights the importance of the spectroscopic investigations performed simultaneously with the transport measurements.

## Conclusions

In summary, the combination of the SERS and conductance measurements was applied to the investigation of the molecular junctions accommodating single NT monomer or dimer. The correlation analysis of the vibrational energy and conductance demonstrated the three different bondings in the NT junctions, i.e., the π–π interaction in the dimer junction, and the through-π and through-space couplings involved in both the monomer and dimer junctions. Importantly, the analysis revealed that the NT junctions with different bondings coexist in the same conductance region, and, consequently, the electronic characterization alone failed to distinguish the two junctions. The theoretical calculations support the experimental findings based on the conductance and vibrational energy behavior in response to the structural changes in the molecular junctions. The analysis using the power density spectra allows an estimation of the flicker noise and proves the electron transport mediated by the π–π interaction. The present study provides the detailed structural identification of the molecular junction of the single dimer of the aromatic compound and unveiled its intrinsic electron-transport properties, indicating that the spectroscopic study simultaneous with the transport measurements is vital in the study of molecular junctions of a single dimer.

## Supporting Information Available

The calculation for vibrational energy of the monomer and dimer junction, Raman spectra of the NT-powder sample and NT-monomer and dimer junction, procedure for the estimation of the flicker noise, two-dimensional conductance-stretch length histogram, relationship between vibrational energy and conductance, calculation for the transmission spectra, distribution of the vibrational energy in the lower conductive region, and evolution of the NT monomer junction (PDF)
