# Supplementary material for: Intermolecular and Electrode-Molecule Bonding in a Single Dimer Junction of Naphthalenethiol as Revealed by Surface-Enhanced Raman Scattering Combined with Transport Measurements
Source: J Am Chem Soc. 2023 Jul 12;145(29):15788–95. doi: 10.1021/jacs.3c02050 (PMC10375526; doi:10.1021/jacs.3c02050)
Supplement: Supplementary file 1 — ja3c02050_si_001.pdf [file ja3c02050_si_001.pdf]

Supporting Information.

Intermolecular and Electrode-Molecule Bonding in a Single Dimer Junction of Naphthalenethiol as Revealed by Surface-Enhanced Raman Scattering Combined with Transport Measurements.

Kanji Homma[a], Satoshi Kaneko*[a], Kazuhito Tsukagoshi[b], Tomoaki Nishino*[a]

[a]*Department of Chemistry, School of Science, Tokyo Institute of Technology, 2-12-1 W4-10 Ookayama, Meguro-ku, Tokyo 152-8551, Japan*
[b]*International Center for Materials Nanoarchitectonics (WPI-MANA), National Institute for Materials Science (NIMS), 1-1 Namiki, Tsukuba, Ibaraki 305-0044, Japan*

* Corresponding authors: S.K. (skaneko@chem.titech.ac.jp), T.N. (tnishino@chem.titech.ac.jp)

Table of Contents

S1. Estimation of flicker noise. S-2
S2. Scaling of flicker noise in a single-molecule junction. S-3
S3. Geometry of the single-molecule junction of the theoretical simulation. S-5
S4. Two-dimensional conductance histogram. S-7
S5. SERS of NT molecular junction and normal Raman spectrum of NT. S-8
S6. SERS signal intensity at the molecular junction. S-11
S7. Relationship between vibrational energy and conductance. S-12
S8. Displacement dependence of Energy for NT dimer. S-13
S9. Distribution of the vibrational energy focusing on the Au/NT dimer/Au junction. S-14
S10. Evolution of the NT monomer junction. S-15
References S-16

S1. Estimation of flicker noise

Figure S1 (a) represents the typical frequency dependence of the noise power during Surface-enhanced Raman scattering (SERS) observation at a single-molecule junction. The noise power density (S_n) follows a power law with the frequency (f) *i.e.* $S_n \propto f^{-\gamma}$. The corresponding histogram gives most probable value of 1.1±0.4, supporting that noise originating from flicker noise observed for molecule junctions.[1-3]

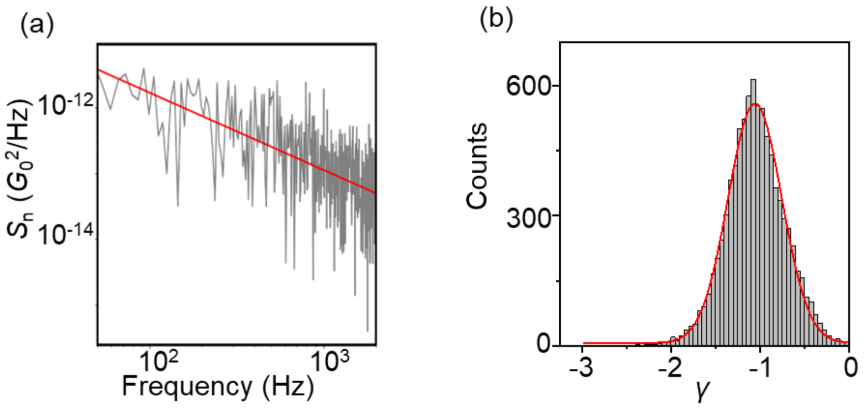

Figure S1. (a) Frequency dependency of the power spectra density (S_n). Red line indicates $S_n \propto f^{-1.2}$. (b) Histogram of γ in the relation $S_n \propto f^{\gamma}$. Red line shows Gaussian fit. The peak center is -1.1±0.4.

S2. Scaling of flicker noise in a single-molecule junction

Before discussion of conductance fluctuation of molecular junction, we mention the expression of the conductance of the junction. The conductance (G) can be derived from the Landauer formular with Breit-Wigner resonance model, as shown equation S1.[4]

$$G = G_0 \frac{\Gamma^2}{(E-\varepsilon_0)^2+\Gamma^2} \approx G_0 \frac{\Gamma^2}{{E_0}^2}, \qquad (E_0 = E\text{-}\varepsilon_0) \tag{S1}$$

, where E represents the energy of the electron, ε_0 represents the molecular orbital energy with respect to the Fermi energy, G_0 represents the quantized unit of electrical conductance ($2e^2/h$) and Γ represents the broadening of the molecular orbital induced by the metal-molecule interaction. Then, the fluctuation of the conductance ($<\Delta G^2>$) can be obtained by the derivative of (S1).

$$< \Delta G^2 > = 4{G_{AVE}}^2 \frac{< \Delta\Gamma^2 >}{{\Gamma_{AVE}}^2} \tag{S2}$$

Assuming that the Γ derived from the interaction was expressed by the product of exponential term and the fluctuation term ($p(z)$).[1]

$$\Gamma = \Gamma_0 \exp(-\beta z) \times p(z) \tag{S3}$$

, where β represents the decay constant. On the condition that fluctuation of p(z) can be expressed by the Gauss function, the fluctuation of Γ can be expressed as follows.[1]

$$< \Delta\Gamma^2 > = \int dz\, {\Gamma_0}^2 e^{-2bz_0} \frac{1}{\sqrt{2\pi\sigma^2}} e^{-\frac{(z-z_0)^2}{2\sigma^2}} - < \Gamma >^2 = < \Gamma >^2 \left[e^{2(\beta\sigma)^2} - 1\right] \tag{S4}$$

By substituting eq. (S4) to eq. (S2) gives eq. (S5)

$$< \Delta G^2 > = 4{G_{AVE}}^2\left[e^{2(\beta\sigma)^2} - 1\right] \tag{S5}$$

The conductance fluctuates depend on the ${G_{AVE}}^2$.

When we assume the dimer junction, we regard the electron transport via the levels that is correlated with the interaction term (δ). Similar to eq.1, the conductance of the junction can be expressed as shown in eq. (S6), assuming the δ is much smaller than E and ε_0.

$$G \approx G_0 \frac{\Gamma^2\delta^2}{{E_0}^4} \tag{S6}$$

The derivative of (S6) gives

$$\Delta G = 2G_{AVE}\left(\frac{\Delta\Gamma}{\Gamma_{AVE}} + \frac{\Delta\delta}{\delta_{AVE}}\right) \tag{S7}$$

,where

$$G_{AVE} = G_0 \frac{{\Gamma_{AVE}}^2 {\delta_{AVE}}^2}{{E_0}^4}$$

Therefore,

$$\Delta G = 2G_{AVE}\frac{\Delta\delta}{\delta_{AVE}} + 2{G_0}^{0.5}{G_{AVE}}^{0.5}\frac{\delta_{AVE}\Delta\Gamma}{{E_0}^2} \tag{S8}$$

Thus, the conductance fluctuation $<\Delta G^2>$ depends on the $(\Delta\delta)^2{G_{AVE}}^2$ and $(\Delta\Gamma)^2 G_{AVE}$. In the case that the fluctuation of the interaction between molecules mainly contributes to conductance fluctuation, $<\Delta G^2> \propto {G_{AVE}}^2$. On the other hand, the case that the fluctuation of the metal-molecule interaction mainly contributes to conductance fluctuation, the $<\Delta G^2> \propto G_{AVE}$. Even in the dimer junction, the both molecule-molecule and metal-molecule interaction can be contributed to the conductance fluctuation, the conductance fluctuation have the value of $1 < <\Delta G^2> < 2$.[5-6]

We then estimated the scaling of the noise power following the previous method for H and L states.[1] We calculated the correlation factor between S_n and G^λ regarding the scaling exponent (λ) as shown in Figure S2. The scaling exponent of the system was determined as the value makes the correlation coefficient zero. Before discussing

the scaling exponent, we have to judge the validity of the data set for the correlation analysis. While even the maximum correlation factor was 0.4 in the H state, that was only 0.1 in the low states. Namely, S_n and G are weakly correlated in the L state, while those show no correlation in the H state. It can be considered that the small conductance window for the analysis in the H state derived from the significant modulation in the binding energy of Au-π interaction hampers the precise estimation of the scaling.

According to previous studies, polycyclic aromatic hydrocarbons such as benzene and naphthalene physisorbed on the Au surface show a variation in the binding energy from 0.5 eV to 1.5 eV. [7-10] Under the room temperature measurement of around 300 K, the molecule and atoms are thermally fluctuated among these states and modulate the Au-Naphthalenethiol (NT) distance, prompting immediate geometrical changes from the H state to the L state. The immediate change from the H state to the L state in the Au-π interaction is validated by the separation dependence of the conductance and as shown in Figure 4. The conductance significantly changes in response to the separation distance. The immediate change from the rigid bond with high conductivity to the weak bond with low conductivity narrows the distribution in the conductance in the H state and broadens that in the L state.

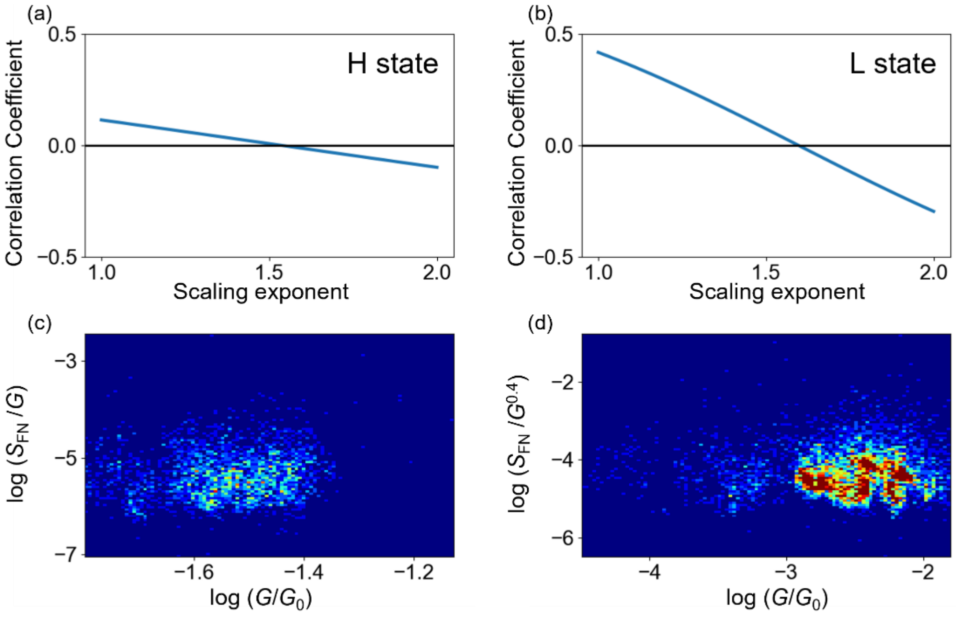

Figure S2. Correlation of the noise power and condutance for H and L staete.

S3. Geometry of the single-molecule junction of the theoretical simulation.

We built the junction structure and calculated the transmission spectra using the Virtual NanoLab - Atomistix Tool Kit package (Quantum ATK ver. 2014.2), which is based on the DFT combined with the nonequilibrium Green's function method.[11] The optimization and transport calculation were performed by the Perdew-Burke-Ernzerhof (PBE) generalized gradient approximation (GGA) exchange-correlation energy functional. The electrode was built from the (111) direction of the periodic gold structure in the ATK package. The top layer consists of three gold atoms for the connection to NT. Thiol atoms were initially set on the hollow site and the geometry was optimized until the maxim force on each atom is less than 0.05 eV/Å. A cutoff energy of 75 Hartree with the 3x3x50 k-point mesh were applied. The basis functions were Single-Zeta polarized for Au, and Double-Zeta polarized for the other atoms. After the optimization, the S-Au distance is 2.5 Å and distance between two naphthalene rings were 3.1-3.4 Å, agreeing with the previous study.[10, 12] To evaluate the electron transport through the NT junction, we constructed the models with a variety of separation distances to find the preferable junction structure in the breaking process of the junction. The number of the models was up to 33 and 58 models for monomer and dimer junctions, respectively. The optimized structure and calculated transmission spectra at the separation were shown in Figures S3 and S4 for Au/NT monomer/Au junction and Au/NT dimer/Au junction.

We, then, rebuilt the junction structure for the vibrational calculation from the optimized structure. We construct the electrode with three gold layers. The structure converted into the Gaussian 16 format, and the junction geometry of the junction was optimized by the density functional theory (DFT) method of CAM-B3LYP with Grimme's D3BJ empirical dispersion correction.[13] The basis function were LanL2DZ for gold, and 6-31G(d) for carbon, hydrogen, and sulfur atoms to include the effect of the dispersion force. The scaling factor was 0.95.[14]

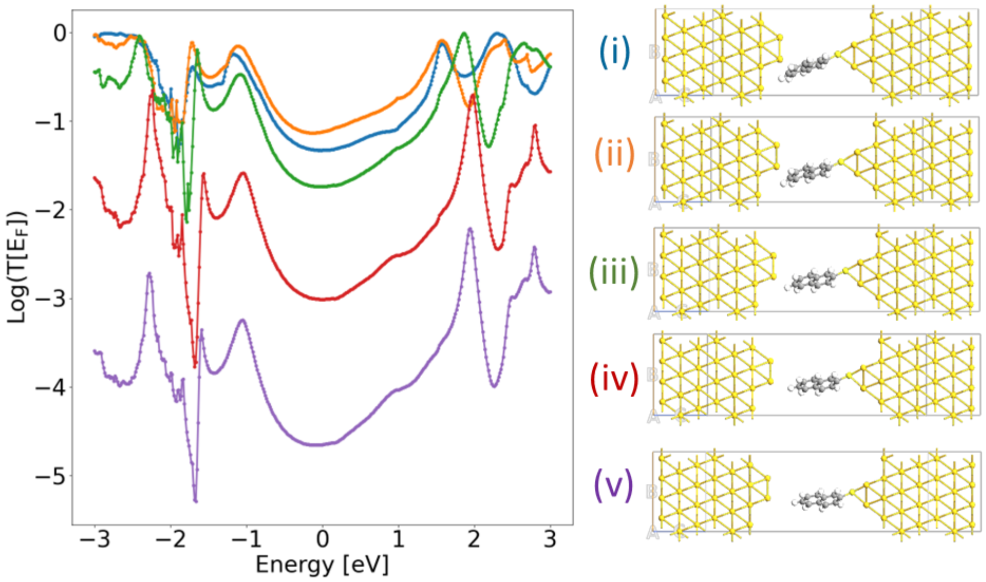

Figure S3. Transmission spectra depending on the separation distance for Au/NT/Au junction.

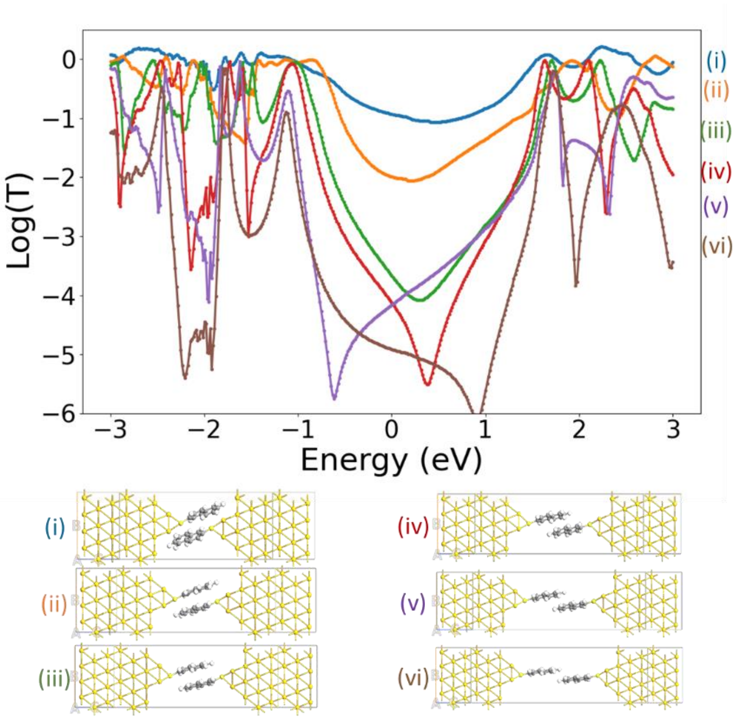

Figure S4. Transmission spectra depending on the separation distance for Au/NT dimer/Au junction.

S4. Two-dimensional conductance histogram.

We confirmed the formation of the NT junction based on the mechanically-controllable break-junction method.[15-17] Figure S5 (a) shows typical conductance traces measured with clean Au (black line) and NT-modified (blue lines) electrodes. In the case of the clean Au electrode, a single distribution appeared at 1 G_0 in the 2D histogram (Figure S5(b), left) complied from the conductance traces, which arises from the Au atomic junction. In contrast, the 2D histogram exhibited cloud-like distributions in the range of $10^{-3.5}$–$10^{-1.5}$ G_0 in the presence of the NT molecules on the electrode (Figure S5(b), right), and these distributions demonstrate the formation of NT molecular junctions. Importantly, the conductance range for the NT molecular junctions observed in the MCBJ study agrees well with the one found in the *I*-*V* measurements simultaneously acquired with SERS (Figure 2(c)). Thus, the results in Figure S5 prove the formation of the NT molecular junctions in the simultaneous SERS and *I*-*V* measurements discussed in the main text.

It is noteworthy that the conductance states of the NT molecular junctions appeared as cloud-like distributions in the 2D histogram (Figure S5(b), right). We ascribed this to insufficient mechanical stability of the NT dimer junction against the external force exerted during the MCBJ process. The binding energy of the NT dimer was estimated to be around 0.35 eV (see Section S7), while in the case of terphenylenedithiol dimer, which afforded distinct distribution in the 2D conductance histogram, the binding energy of approximately 2 eV was found.[6] Despite the blurred appearance of the NT molecular junctions in the 2D conductance histogram, their conductive states were clearly resolved in the 2D mapping of the conductance and the Raman shift (Figure 3(a)). This difference arises most probably due to the absence of the external force in the simultaneous SERS and *I*-*V* measurements, which involves no mechanical displacement of the electrode.

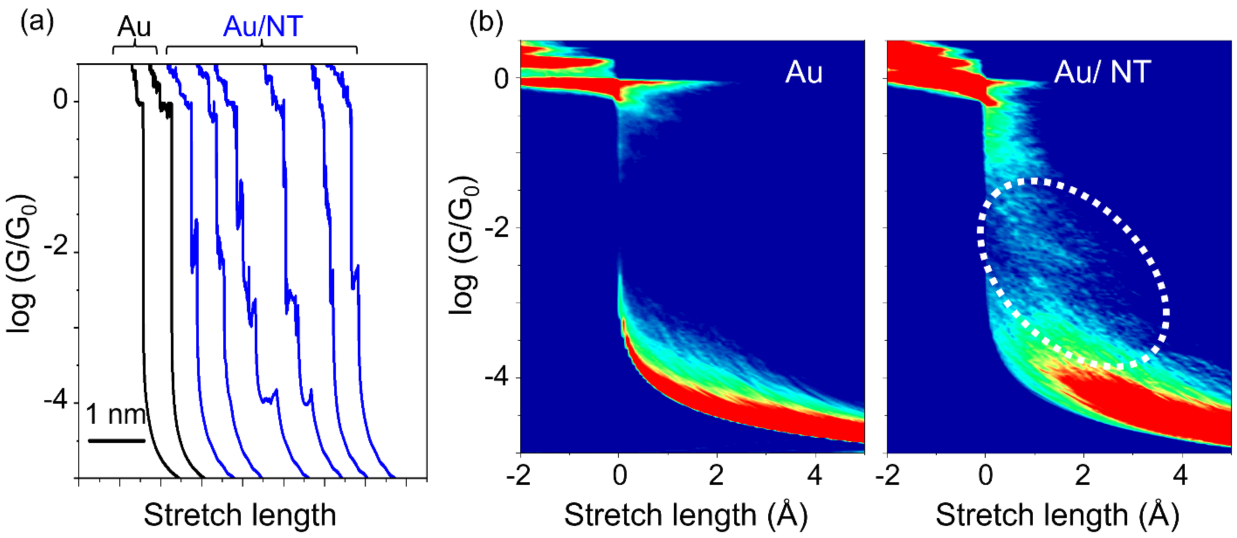

Figure S5. MCBJ measurements for the NT junction. (a) Typical conductance traces obtained with clean Au (black line) and NT-modified (blue lines) electrodes. (b) 2D conductance histogram obtained with clean Au (left) and NT-modified (right) electrodes. The left and right histograms were constructed with 2169 and 2002 traces, respectively.

S5. SERS of NT molecular junction and normal Raman spectrum of NT

Figure S6 represents SERS spectra of Au/NT dimer/Au junction, histogram of the vibrational energy, Raman spectra of the powder sample and the calculated spectra of Au/NT dimer/Au junction. The SERS spectra qualitatively resembles with the powder sample and theoretical simulation, allowing to the vibrational mode observed around 830 cm^{-1}, 1070, 1380, and 1620 cm^{-1}, were assigned to the vibrational mode marked (1), (3), (6), and (10) in the calculated spectrum. The displacement of the atoms for each vibrational mode were displayed in Figure S7. We show the example of the SERS spectra and corresponding *I-V* curves for the high and low conductive states, respectively, in Figure S8.

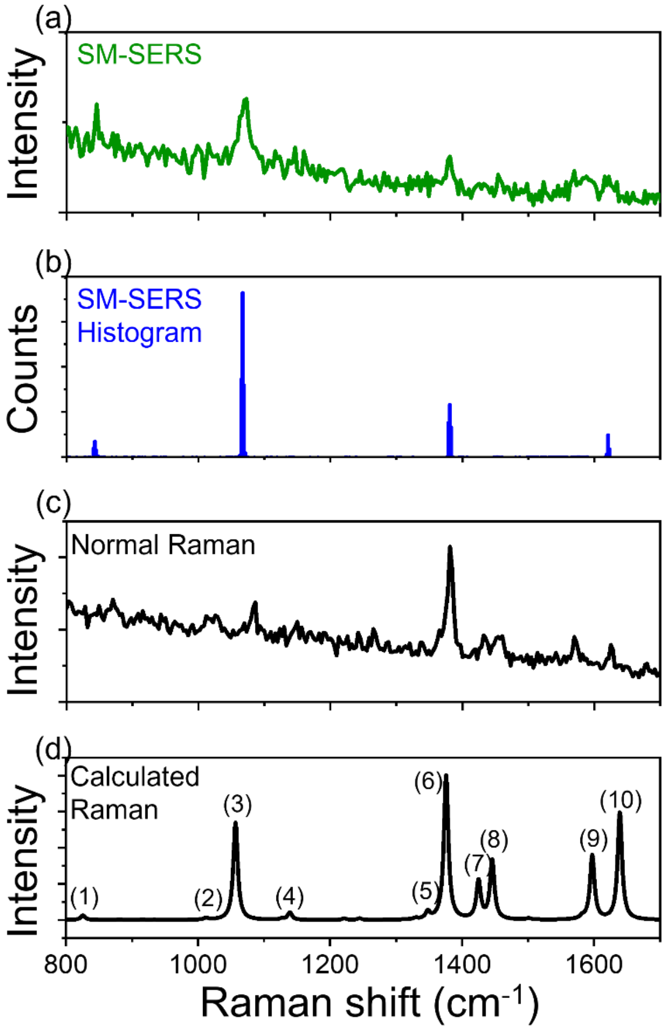

Figure S6. (a) Typical example of SERS spectrum of single-molecule junction. (SM-SERS). (b) Histogram of the vibrational energy obtained by the fitting of the SM-SERS. (c) Normal Raman obtained by measuring the powder NT. (d) The theoretically calculated NT molecule.

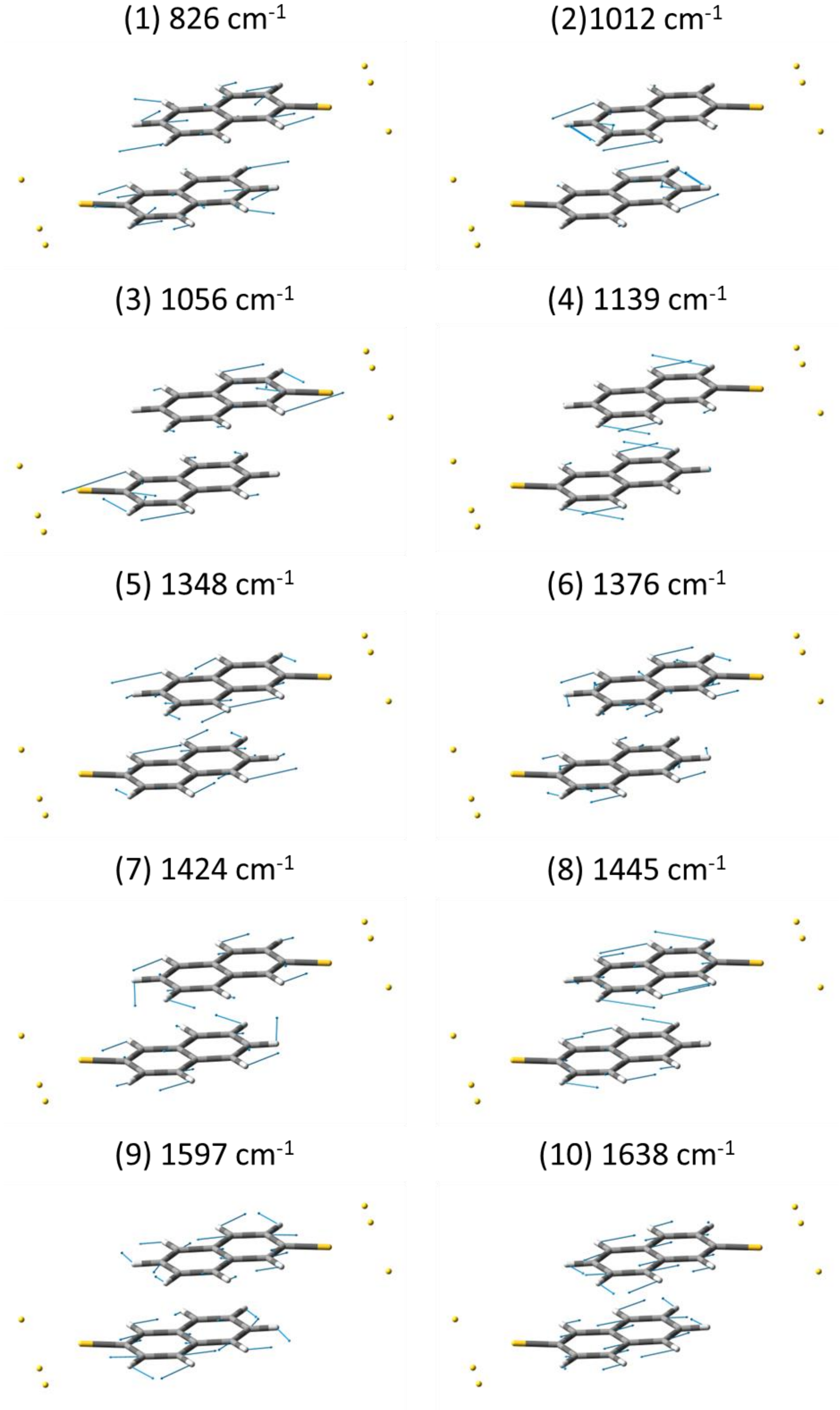

Figure S7. Vibrational energy and displacement vector of the vibrational modes of Au/NT dimer/Au junction focusing on the relevant region of the junction. NT represent as the wire frame. The yellow sphere represents gold atom.

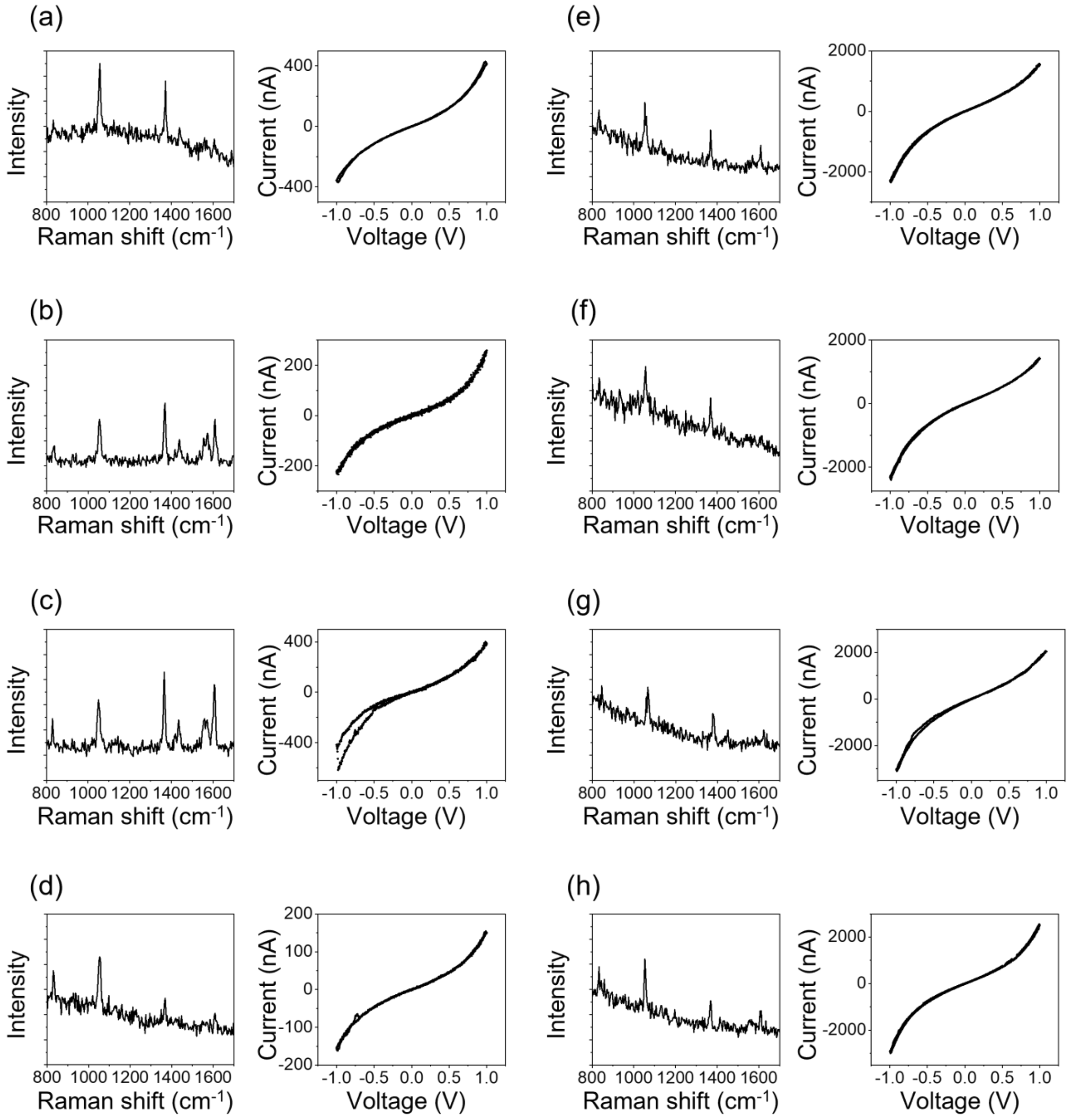

Figure S8. Example of the SERS and *I-V* response obtained for the NT molecular junction. (a-d) for the high-conductive region. (e-h) for the low conductive region.

S6. SERS signal intensity at the molecular junction.

In the single-molecular junction, SERS signal was enhanced by the electromagnetic field effect and charge transfer effect, though the electromagnetic field effect also affects the molecule at the relevant of the junction on the gold surface, the molecule bridging over metal nanogap obtains additional charge transfer effect, leading to the strong SERS signal in the molecular junction.[18-21] Figure S9 illustrates pairs of the simultaneously measured SERS spectrum and *I-V* curve obtained with the NT-modified electrodes. For Figure S9(a), the conductance was determined to be 10^{-3} G_0 based on the *I-V* curve. This value agrees with the conductance of the NT single-molecule junction (Section S4), demonstrating the presence of the molecular junction. In contrast, for Figure S9(b), the conductance was as low as 10^{-6} G_0 significantly lower than the single-molecule conductance of NT, and the molecular junction was absent during the measurements. Comparison between the SERS spectra in Figure S9 reveals that the SERS intensity of the molecular junction was significantly enhanced compared with that of disconnected states. Figure S10 shows the relationship between the SERS intensity and the conductance. It was confirmed that the SERS intensity significantly increased. We also observed the blinking of the SERS signal depending on the conductance value and the intensity of the ring breathing mode was enhanced in the region of $10^{-3.8}$–10^{-2} G_0 corresponding to the conductance of the molecular junction (Figure S10)

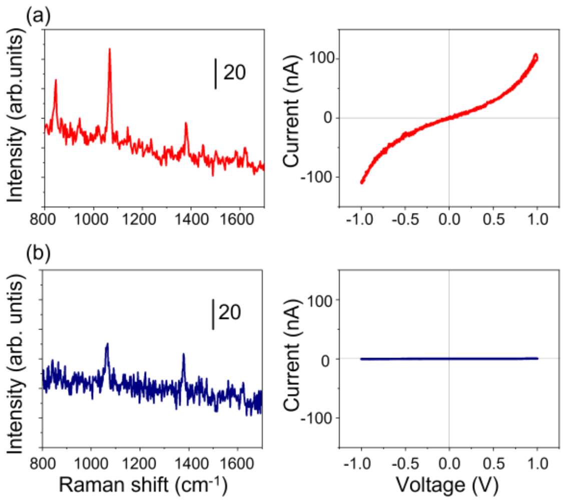

Figure S9. (a, b) Simultaneously measured SERS spectra and *I-V* curves.

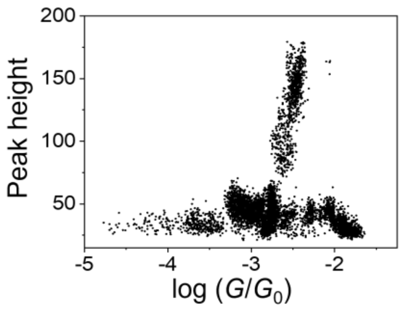

Figure S10. Relationship between conductance and peak hight of the ring breathing mode of NT.

S7. Relationship between vibrational energy and conductance.

The relationship between the vibrational energy of C-H bending mode ($\nu_{\text{C-H bending}}$) and conductance in a log scale (log (G/G_0)) was depicted in Figure S11. The L1 and L2 states were clearly separated into two distinctive regions. The positive correlation in the vibrational energy between ring breathing mode ($\nu_{\text{ring breathing}}$) and C-H bending mode supports the consistency of the spectroscopic observation. Some states, especially in the high conductive region, cannot be observed, because the peak intensity is different among the vibrational and small intensity is under the detection limit.

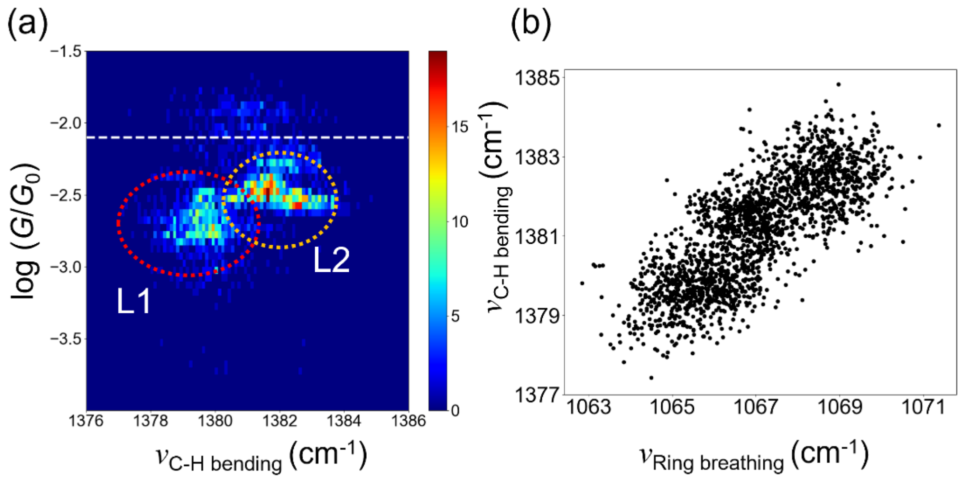

Figure S11. (a) Two-dimensional histogram regarding conductance and vibrational energy of C-H bending mode observed by the SERS measurement of NT single-molecule junction. The number of bins were 100 and 50 for *x* and *y* axis. (b) Relationship of the vibrational energy between Ring breathing mode (observed around 1380 cm^{-1}) and C-H bending mode (observed around 1067 cm^{-1}).

S8. Displacement dependence of Energy for NT dimer

We calculated the energy of the NT dimer depending on the displacement along the long axis of the dimer. The geometric optimization was performed in Gaussian 16 format by the method of CAM-B3LYP with Grimme's D3BJ empirical dispersion correction. The basis function was 6-311++G(d,p). We calculated the energy of the dimer depending on the displacement by shifting the top monomer from 0 to 8 Å, with an interval step of 0.4 Å with fixing the S atoms (Figure S8). The binding energy of the dimer was estimated to be 350 meV by subtracting the energy at the separated state from the that fully overlapped state. The estimated binding energy agrees well with the previous study.[10, 22]

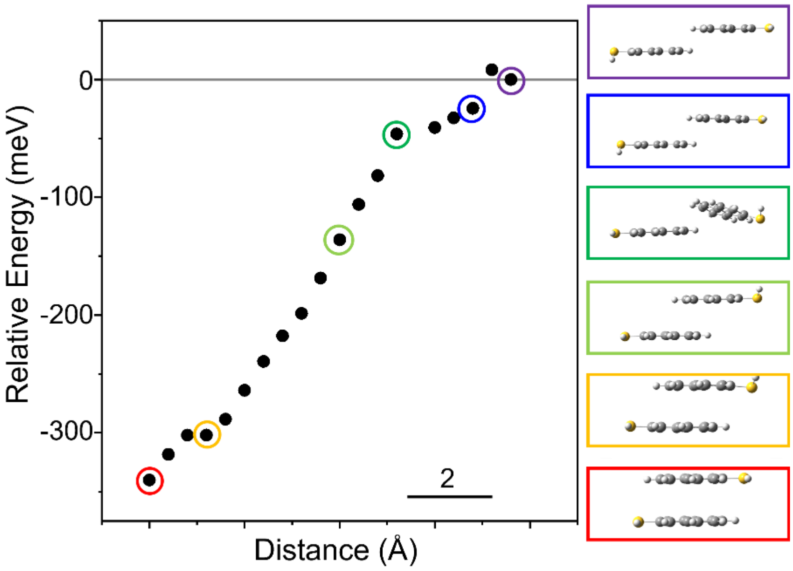

Figure S12. Displacement dependence of energy of NT dimer. Inset represents the optimized structure at the corresponding-colored circle.

S9. Distribution of the vibrational energy focusing on the Au/NT dimer/Au junction.

The two-dimensional histogram of conductance and Raman shift of ring breathing mode focusing on the L-state was depicted in Figure S13. Even lower conductance region than the L2 state, the distribution of the vibrational mode contains at least two components whose peak centers are 1066 cm^{-1} and 1067 cm^{-1}. The smaller vibrational energy might originate from dimer junction and lager one might originate from disconnected states.

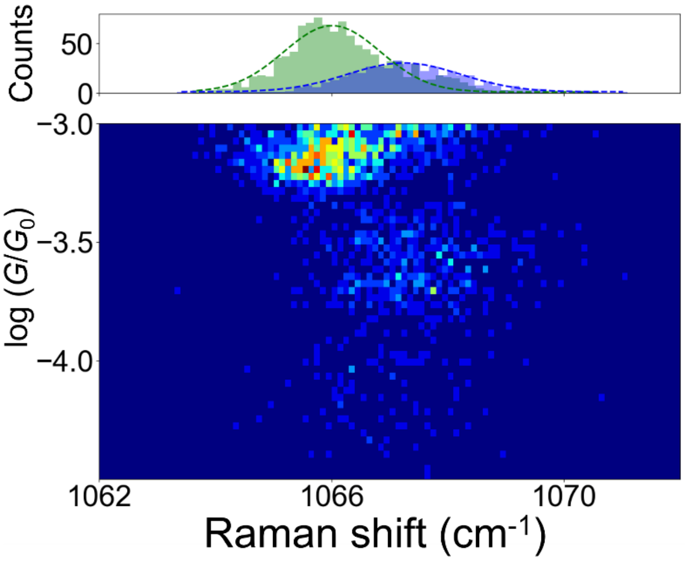

Figure S13. Two-demensional histogram of condutance and Raman shift.

S10. Evolution of the NT monomer junction.

DFT calculations were performed to investigate the evolution of the NT monomer junction as the increase in the gap width between the electrodes (Figure S15). The computational details were the same as those employed for the NT dimer (Section S8). The total energy of the NT monomer junction was found to monotonically increase as the gap width increases. The slope of the energetic changes around the junction structure with the lowest energy is 174 meV/Å. This value is substantially larger than the corresponding value found for the NT dimer (48 meV/Å, see Section S8). The steep energetic increase indicates that the monomer junction adopts very limited structure leading to the very limited variation in the junction conductance.

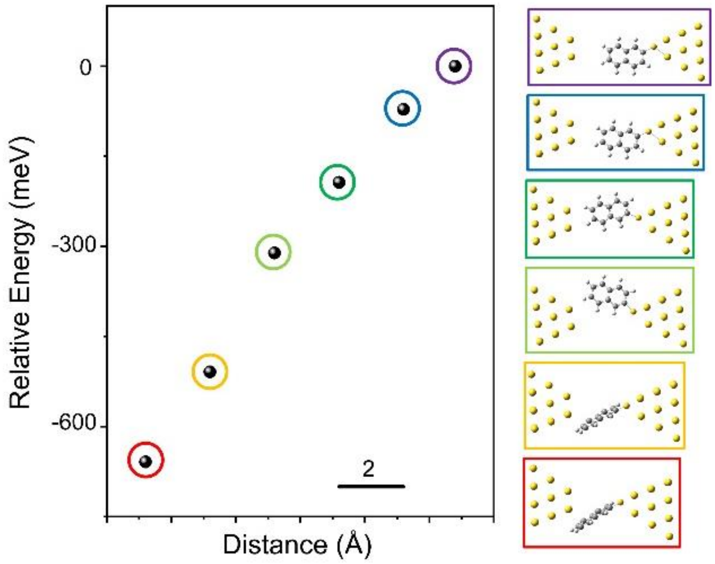

Figure S15. Evolution of the energy of the NT monomer junction calculated by DFT.

References

(1) Adak, O.; Rosenthal, E.; Meisner, J.; Andrade, E. F.; Pasupathy, A. N.; Nuckolls, C.; Hybertsen, M. S.; Venkataraman, L., Flicker Noise as a Probe of Electronic Interaction at Metal-Single Molecule Interfaces. *Nano Lett.* **2015,** *15*, 4143-4149.

(2) Magyarkuti, A.; Adak, O.; Halbritter, A.; Venkataraman, L., Electronic and Mechanical Characteristics of Stacked Dimer Molecular Junctions. *Nanoscale* **2018,** *10*, 3362-3368.

(3) Diaz-Alvarez, A.; Higuchi, R.; Sanz-Leon, P.; Marcus, I.; Shingaya, Y.; Stieg, A. Z.; Gimzewski, J. K.; Kuncic, Z.; Nakayama, T., Emergent Dynamics of Neuromorphic Nanowire Networks. *Sci. Rep.* **2019,** *9*, 14920.

(4) Cuevas, J. C.; Scheer, E., *Molecular Electronics: An Introduction to Theory and Experiment*. World Scientific Publishing Co.Pte. Ltd.: Singpore, 2017.

(5) Li, X.; Wu, Q.; Bai, J.; Hou, S.; Jiang, W.; Tang, C.; Song, H.; Huang, X.; Zheng, J.; Yang, Y.; Liu, J.; Hu, Y.; Shi, J.; Liu, Z.; Lambert, C. J.; Zhang, D.; Hong, W., Structure-Independent Conductance of Thiophene-Based Single-Stacking Junctions. *Angew. Chem. Int. Ed.* **2020,** *132*, 3306-3312.

(6) Tang, Y.; Zhou, Y.; Zhou, D.; Chen, Y.; Xiao, Z.; Shi, J.; Liu, J.; Hong, W., Electric Field-Induced Assembly in Single-Stacking Terphenyl Junctions. *J. Am. Chem. Soc.* **2020,** *142*, 19101-19109.

(7) Li, C.; Chen, Z.; Huang, Y.; Zhang, Y.; Li, X.; Ye, Z.; Xu, X.; Bell, S. E. J.; Xu, Y., Uncovering Strong π-Metal Interactions on Ag and Au Nanosurfaces under Ambient Conditions via in-situ Surface-Enhanced Raman Spectroscopy. *Chem* **2022,** *8*, 2514-2528.

(8) Liu, W.; Tkatchenko, A.; Scheffler, M., Modeling Adsorption and Reactions of Organic Molecules at Metal Surfaces. *Acc. Chem. Res.* **2014,** *47*, 3369-3377.

(9) Molina, L. M.; López, M. J.; Alonso, J. A., Interaction of Aromatic Molecules with Small Gold Clusters. *Chem. Phys. Lett.* **2017,** *684*, 91-96.

(10) Saeki, M.; Akagi, H.; Fujii, M., Theoretical Study on the Structure and the Frequency of Isomers of the Naphthalene Dimer. *J. Chem. Theory Comput.* **2006,** *2*, 1176-1183.

(11) Smidstrup, S.; Markussen, T.; Vancraeyveld, P.; Wellendorff, J.; Schneider, J.; Gunst, T.; Verstichel, B.; Stradi, D.; Khomyakov, P. A.; Vej-Hansen, U. G.; Lee, M. E.; Chill, S. T.; Rasmussen, F.; Penazzi, G.; Corsetti, F.; Ojanpera, A.; Jensen, K.; Palsgaard, M. L. N.; Martinez, U.; Blom, A.; Brandbyge, M.; Stokbro, K., QuantumATK: An Integrated Platform of Electronic and Atomic-Scale Modelling Tools. *J. Phys. Condens. Matter* **2020,** *32*, 015901.

(12) Feng, A.; Zhou, Y.; Al-Shebami, M. A. Y.; Chen, L.; Pan, Z.; Xu, W.; Zhao, S.; Zeng, B.; Xiao, Z.; Yang, Y.; Hong, W., Sigma-Sigma Stacked Supramolecular Junctions. *Nat. Chem.* **2022,** *14*, 1158-1164.

(13) Grimme, S.; Ehrlich, S.; Goerigk, L., Effect of the Damping Function in Dispersion Corrected Density Functional Theory. *J. Comp. Chem.* **2011,** *32*, 1456-1465.

(14) Arı, H.; Özpozan, T.; Büyükmumcu, Z.; Akın, N.; İlhan, İ. Ö., Synthesis, Spectral and Theoretical (DFT) Investigations of 4,6-Diphenyl-6-hydroxy-1-{[(1Z)-1-phenyl ethylidene] Amino}tetrahydropyrimidine-2(1H)-one. *J. Mol. Struct.* **2022,** *1250*, 131820.

(15) Kamenetska, M.; Koentopp, M.; Whalley, A. C.; Park, Y. S.; Steigerwald, M. L.; Nuckolls, C.; Hybertsen, M. S.; Venkataraman, L., Formation and evolution of single-molecule junctions. *Phys Rev Lett* **2009,** *102*, 126803.

(16) Fukuzumi, R.; Buerkle, M.; Li, Y.; Kaneko, S.; Li, P.; Kobayashi, S.; Fujii, S.; Kiguchi, M.; Nakamura, H.; Tsukagoshi, K.; Nishino, T., Water Splitting Induced by Visible Light at a Copper-Based Single-Molecule Junction. *Small* **2021,** *17*, e2008109.

(17) Evers, F.; Korytár, R.; Tewari, S.; van Ruitenbeek, J. M., Advances and Challenges in Single-Molecule Electron Transport. *Rev. Mod. Phys.* **2020,** *92*, 035001

(18) Liu, Z.; Ding, S. Y.; Chen, Z. B.; Wang, X.; Tian, J. H.; Anema, J. R.; Zhou, X. S.; Wu, D. Y.; Mao, B. W.; Xu, X.; Ren, B.; Tian, Z. Q., Revealing the Molecular Structure of Single-Molecule Junctions in Different Conductance States by Fishing-Mode Tip-Enhanced Raman Spectroscopy. *Nat. Commun.* **2011,** *2*, 305.

(19) Kaneko, S.; Murai, D.; Marques-Gonzalez, S.; Nakamura, H.; Komoto, Y.; Fujii, S.; Nishino, T.; Ikeda, K.; Tsukagoshi, K.; Kiguchi, M., Site-Selection in Single-Molecule Junction for Highly Reproducible Molecular Electronics. *J. Am. Chem. Soc.* **2016,** *138*, 1294-1300.

(20) Liu, S.; Hammud, A.; Wolf, M.; Kumagai, T., Atomic Point Contact Raman Spectroscopy of a Si(111)-7

x 7 Surface. *Nano Lett.* **2021,** *21*, 4057-4061.
(21) Park, W. H.; Kim, Z. H., Charge Transfer Enhancement in the SERS of a Single Molecule. *Nano Lett.* **2010,** *10*, 4040-4048.
(22) Zhang, C.; Cheng, J.; Wu, Q.; Hou, S.; Feng, S.; Jiang, B.; Lambert, C. J.; Gao, X.; Li, Y.; Li, J., Enhanced π-π Stacking between Dipole-Bearing Single Molecules Revealed by Conductance Measurement. *J. Am. Chem. Soc.* **2023,** *145*, 1617-1630.
